# Right care, right time, right place: improving outcomes for people with spinal cord injury through early access to intervention and improved access to specialised care: study protocol

**DOI:** 10.1186/s12913-014-0600-7

**Published:** 2014-12-05

**Authors:** James M Middleton, Lisa N Sharwood, Peter Cameron, Paul M Middleton, James E Harrison, Doug Brown, Rod McClure, Karen Smith, Sandy Muecke, Sarah Healy

**Affiliations:** The University of Sydney, Sydney, Australia; Department of Preventive Medicine, Monash University, Melbourne, Australia; John Walsh Centre for Rehabilitation Research, Sydney, Australia; Discipline of Emergency Medicine, University of Sydney, New South Wales, Australia; Research Centre for Injury Studies, Flinders University, South Australia, Australia; The Spinal Research Institute, Melbourne, Australia; Harvard School of Public Health, Harvard Injury Control Research Centre, Boston, USA; Ambulance Victoria, Research and Evaluation, Melbourne, Australia; NSW Ambulance, New South Wales, Australia; Distributed Research in Emergency and Acute Medicine (DREAM) Collaboration, Sydney, Australia; University Western Australia, Perth, Australia

**Keywords:** Acute traumatic spinal cord injury, Clinical pathways, Patient flow, Trauma systems, Access to specialist care, Quality of care, Outcomes

## Abstract

**Background:**

Traumatic spinal cord injury is a devastating condition impacting adversely on the health and wellbeing, functioning and independence, social participation and quality of life of the injured person. In Australia, there are approximately 15 new cases per million population per year; economic burden estimates suggest 2 billion dollars annually. For optimal patient outcomes expert consensus recommends expeditious transfer (“<24 hours of injury”) to a specialist Spinal Cord Injury Unit, where there is an interdisciplinary team equipped to provide comprehensive care for the many and complex issues associated with traumatic spinal cord injury. No study of this patient population has been undertaken, that assessed the extent to which care received reflected clinical guidelines, or examined the patient journey and outcomes in relation to this. The aims of this study are to describe the nature and timing of events occurring before commencement of specialist care, and to quantify the association between these events and patient outcomes.

**Methods and design:**

The proposed observational study will recruit a prospective cohort over two years, identified at participating sites across two Australian states; Victoria and New South Wales. Included participants will be aged 16 years and older and diagnosed with a traumatic spinal cord injury. Detailed data will be collected from the point of injury through acute care and subacute rehabilitation, discharge from hospital and community reintegration. Items will include date, time, location and external cause of injury; ambulance response, assessments and management; all episodes of hospital care including assessments, vital signs, diagnoses and treatment, inter-hospital transfers, surgical interventions and their timing, lengths of stay and complications. Telephone follow-up of survivors will be conducted at 6, 12 and 24 months.

**Discussion:**

There is limited population level data on the effect of delayed commencement of specialist care (>24 hours) in a Spinal Cord Injury Unit. Examining current health service and clinical intervention pathways in this Australian population-based sample, in relation to their outcomes, will provide an understanding of factors associated with patient flow, resource utilisation and cost, and patient and family quality of life. Barriers to streamlined effective early-care pathways and facilitators of optimal treatment for these patients will be identified.

## Background

While there is rapidly growing understanding of the underlying neurobiology, and promising advances in the development of novel therapeutics for central nervous system repair and regeneration [1], traumatic spinal cord injury (TSCI) remains a devastating, irreversible condition. The leading causes of TSCI are motor vehicle crashes, falls, sporting accidents and violence, which affects between 12.1 per million to 57.8 per million new people worldwide each year [2]. In Australia, around 300 people (approximately 15 cases per million population) sustain a new acute TSCI each year [3]. Injuries most often occur at a young age (30% in 15-24 year age group), although trends show a marked increase in the average age at injury with a bimodal distribution and growing numbers of older persons [4,5]. Acute survival has improved with advances in emergency medicine and intensive care management, and life expectancy following TSCI now approaches that of the general population for all but the most severely impaired individuals [6]. Life after TSCI, however, is often associated with increased secondary health complications, activity limitations, reduced community participation and quality of life [7-11]. These factors have a major impact not only on the injured person, but on their family and the community. Despite a relatively low incidence, the human, social and long term financial costs associated with TSCI are extremely high, with the lifetime cost in Australia for a person with paraplegia estimated at $5 million and for tetraplegia at $9.5 million [12].

The first 24 hours after any traumatic injury are acknowledged as the most critical for survival, requiring prompt recognition, early evaluation and appropriate management in a suitable setting [13,14] to achieve best outcomes. Expert consensus [15] recommends expeditious transfer of the suspected TSCI patient (within 24 hours of injury) to a specialised spinal cord injury unit (SCIU) equipped to provide comprehensive, state-of-the-art care by an expert interdisciplinary team. Expeditious transfer enables more rapid diagnosis and intervention with time-critical neurosurgical procedures [16-18] and emerging pharmacologic therapies [19] that can enhance preservation (neuroprotection) and possible recovery of neurological function, as well as prevent secondary complications. Delays in reaching specialist care are known to increase the occurrence of complications such as avoidable pressure injuries, urinary tract infections, respiratory problems and contractures; potentially increasing morbidity and length of stay, delaying or impeding rehabilitation, and adversely affecting long-term wellbeing, function and independence related outcomes [20-27].

In a recent pilot study [28] retrospectively linking pre-hospital and hospital outcome data for a cohort of patients with TSCI between 2004 and 2008 in New South Wales (NSW), people injured in non-metropolitan and rural regions generally reached specialist care (in a SCIU) in Sydney more quickly than those initially transported by ambulance to a major (non-SCIU) metropolitan trauma service. Multiple trauma patients were more likely to experience delays than those with isolated TSCI; however, delays of more than 24 hours to reach an SCIU were also demonstrated in patients with TSCI alone. These delays were associated with 2.5 times greater likelihood of having a secondary complication, such as deep vein thrombosis (DVT) or pressure injury (95% CI 1.51–4.17, p < 0.001) [28]. Barr [29] reported not only a significantly increased complication risk on admission with such delays, but equally, significantly longer lengths of hospital stay for patients with complications on admission. Early admission to a SCIU has demonstrated reduced hospital length of stay by 30%, a three-fold reduction in the rate of pressure ulcers [30] and reduced DVT incidence (2% vs 26%) [31].

Ideal treatment depends on having an effective and coordinated health care system capable of recognising and treating all patients with suspected TSCI as medical emergencies, employing spinal precautions and rapidly and directly transporting them to a SCIU [32]. Currently, there are limited population level data on the, size and nature of the effect on outcomes of delayed commencement of specialist care in SCIU, and the role other factors play in the early care period after SCI onset. Data are also limited about the journey of a patient with suspected TSCI from the time of injury to definitive care; the specific practices and processes that cause delay, and their degree of impact on achievement of optimal outcomes. The extent to which these factors are amenable to modification remains unknown.

Therefore, the overall aim of this project is to describe, in the Australian states of NSW and Victoria: (a) the clinical journey of people with confirmed TSCI (from injury to definitive diagnosis and specialised treatment in a SCIU, and (b) a range of outcomes for people with confirmed TSCI (to at least 12 months and up to 24 months).

The data obtained will be used to quantify associations between aspects of the clinical journey and patient outcomes, identifying factors associated with health outcomes and well-being, as well as barriers to and facilitators of expeditious access to specialist SCIU care. Overlaying patient flow data onto a detailed environmental scan of health service infrastructure (e.g. designation of hospitals, ward types, staffing profile, 24 hour availability of MRI scanner, dedicated operating theatre for trauma/spinal injury cases), policies, protocols, care processes and resource utilization across both states, will enable analysis of the quality, sufficiency and efficiency of healthcare delivery. The extent, to which the poorer patient outcomes previously identified with delayed access to specialist care holds true at the population level, will be measured.

The study questions implicit in the aim are stated here:

1. In what proportion of cases does duration from injury to commencement of specialist care in SCIU comply with the recommended maximum of 24 hours?
2. Do outcomes differ according to duration from injury to commencement of care in SCIU, and does this confirm the choice of <24 hours as the recommended target?
3. What potentially modifiable factors during the period from injury to commencement of care in SCIU are associated with duration > =24 hours and/or with poorer outcomes?

The study will result in ongoing collaborations, in particular with clinicians in North America, to progress internationally harmonised efforts to understand and measure performance across clinical pathways for TSCI patients, and model patient outcomes to predict impacts of policy or practice change. Recent work in Canada has used simulation modelling to facilitate the design of a tool, ACT Model (Version 1.0), which informs clinical and administrative decisions in TSCI care [33]. This tool is able to simulate TSCI patient flow through a health care system and test “what-if” policy scenarios (e.g. triaging to acute care hospitals, implementing recommendations regarding timing of surgery, reduction in secondary complications) to predict both short and long-term outcomes including life expectancy; health service utilization and costs; and health status and wellbeing following TSCI. It is anticipated that the ACT Model will utilise data from this current study to assist in understanding the complexity of health service pathways and their impact on TSCI patient outcomes in Australia and internationally.

## Methods and design

A prospective cohort study will be used to answer the study questions, with the study subjects being: all cases with a traumatic SCI diagnosis in the Australian states of New South Wales (NSW) and Victoria over a two-year period, aged 16 years or older at onset. The anticipated sample size inclusive of both States is approximately 300 patients, based on incident data for NSW and VIC combined over the previous 10 years, which has averaged approximately 150 cases per year. The configuration of Major Trauma Services and their relation to the specialist SCIUs differs between NSW and Victoria, allowing for comparison of triage protocols, transport times, bypass and inter-hospital transfer policies and care protocols.

### Recruitment

**Figure 1:**
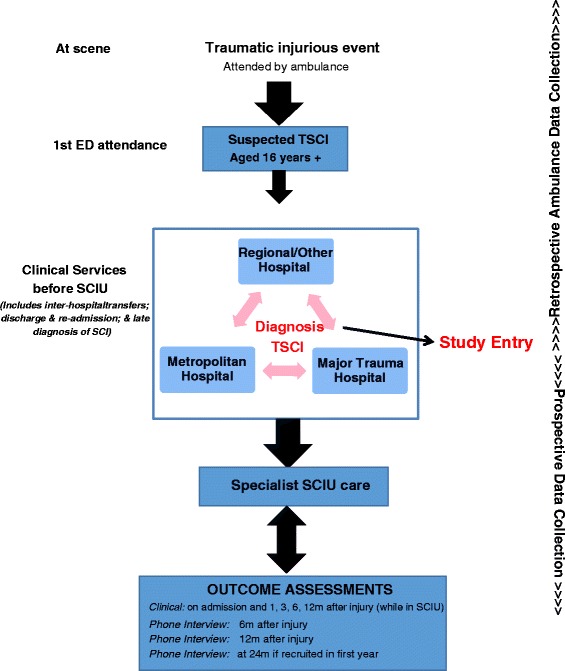
**Schematic diagram of case flows and progression over time.**

Participants will be prospectively identified from any participating site (Major Trauma Services across the State of NSW, specialist SCIUs) (Figure 1). All patients with a possible spinal cord injury due to a traumatic event will be included as potential cases until clinically cleared of spinal cord damage using a current spinal clearance protocol incorporating National Emergency X-Radiography Utilization Study (NEXUS) criteria [34], CT/MRI imaging and consultant review (Alfred Spinal Clearance Management Protocol www.alfred.org.au/Assets/Files/SpinalClearanceManagementProtocol_External.pdf).

From all potential participants identified, those included will have a traumatic spinal cord injury defined as a sudden loss of voluntary muscle strength, sensation and autonomic functions below the level of injury, which will vary depending on neurological level of injury and extent of impairment, but must include altered sacral sensation, in line with international standards for classification. Furthermore, the injury must result in persisting impairment (i.e. not just a concussion) after emergence from neurogenic shock, which generally occurs within the first 24-72 hours after injury. The inclusion criteria are listed below:

1. Traumatic mechanism of injury, including high energy trauma, axial loading to head (e.g. diving, rugby accidents), low energy falls, other blunt or penetrating trauma.

- Focal neurological deficit
- Midline spinal tenderness
- Altered level of consciousness (GCS<15)
- Intoxication
- Painful distracting injuries (eg. Multi-trauma)

2. Positive NEXUS criteria (cervical spine (C-spine)) until cleared, as follows:

NB. Criteria have only been validated for C-spine.

3. Altered neurology on clinical examination, including the presence of:

- Neurogenic (‘spinal’) shock (after exclusion of hypovolemic shock due to blood loss) with lowered systolic blood pressure (eg. <70 mmHg) being associated with bradycardia (< 60 bpm)
- Abnormal neurological signs (such as diaphragmatic breathing using accessory muscles, altered sensation, motor weakness, altered reflex responses – flaccid paralysis during ‘spinal shock’ phase, plantar response).
- Altered bowel/bladder/sacral functions (eg. urinary retention, priapism)

4. Abnormal imaging, such as with MRI scan or multi-slice CT scan.

### Data sources and collection methods

Research Officers will collect medical record data from all confirmed cases of TSCI, who do not withdraw (opt out) (refer to Ethics, below). They will liaise with the Trauma Coordinators at each site for notification of any admission of an eligible TSCI patient to that service.

The data source and collection methods that the Research Officer will use for each item are detailed below, separated for hospital, ambulance and follow-up data:

#### Hospital

1. Injury details (including associated head, chest, intra-abdominal, long bone/pelvic fractures and vascular injuries or coexisting diagnoses such as rheumatoid arthritis, ankylosing spondylitis and spinal stenosis) from medical records and ward discharge summary or Intensive Care Unit (ICU) discharge summary.
2. Review of imaging (X-ray, CT and MRI scans) by an independent radiologist, blinded to the original finding, to quantify extent of spinal canal encroachment, and other radiologic features to correlate with injury epidemiology and outcomes.
3. Earliest recorded classification of spinal cord dysfunction, with documentation of neurological level of injury (NLI) and extent of impairment specified using the International Standards of Neurological Classification for SCI (ISNCSCI) [35] formerly known as American Spinal Injury Association (ASIA) Classification (for all grades of impairment other than grade E), ascertained from the patient’s medical records at discharge.
4. Emergency Department (ED) assessment, including record of vital signs, level of consciousness (GCS), resuscitation, diagnosis and treatment from the patients’ medical record and discharge summary.
5. Inter-hospital transfer dates, times, locations. Delays in referral to specialist care will be documented and investigated.
6. Details of surgical procedure/s to decompress and/or stabilise spine (including date and time performed) collected from the patients’ medical record.
7. Complications, including type, severity and timing of surgical wound infections, bleeding, pressure injuries, pneumonia, thromboembolic disease, urinary tract infection, will be identified from patients’ medical record with supporting evidence from investigations (e.g. Pathology reports or imaging results).
8. Dates of all relevant admissions and separations; as such calculated length of stay (LoS) (total in-hospital and ICU). Discharge destinations for each separation will be collected from the patient’s medical records and discharge summary.

#### Ambulance

Patients identified with confirmed TSCI will have details forwarded to ambulance services in both states for data retrieval. The ambulance services will provide information about date, time, location and external cause of injury, response, treatment and transport intervals, patient disposition at the scene of the injury, interventions used at the scene, spinal precautions and other measures used during transfer to acute care, date and time of arrival at hospital, and time to patient hand-over in ED.

#### Follow-up outcomes

Computer Assisted Telephone Interviews (CATI) will be conducted at 6, 12 and 24 months (for those injured during first year of study) following injury event, using validated tools described below.

### Outcome measures

Analysis will focus on the nature and timing of events occurring before admission to the SCIU and their relationship with the outcomes of interest, as assessed by:

- Extent of neurological recovery documented by improvements in NLI, motor and sensory scores, and impairment grade recorded at the earliest possible time and on discharge from hospital on completion of rehabilitation, using ISNCSCI (formerly ASIA) Classification Standards [35];
- Presence, severity and timing of complications in hospital including location and grade of pressure injuries, pneumonia confirmed on chest XR/CT scan, DVT confirmed by Doppler or Venogram, pulmonary embolism shown on Ventilation-Perfusion scintigraphy or CT Pulmonary Angiogram, contractures with reduced range of motion, wound infections with swab results, urosepsis with urine microscopy, culture and sensitivities, hardware failure and surgical revision on operative reports.
- The Spinal Cord Injury Secondary Conditions Scale [36], a 16-item scale representing problems in the areas of skin, musculoskeletal, pain, bowel/bladder and cardio-vascular function;
- Activity limitation (Functional Independence Measure™ (FIM) score [37,38], Spinal Cord Independence Measure [39] for ambulation);
- LoS in ICU and the definitive treatment hospital until discharge to usual residence, rehabilitation or new place of abode;
- Extent of recovery and Health-Related Quality of Life (HRQoL), assessed by: Glasgow Outcome Scale-Extended (GOS-E)[40], EQ-5D™ [41], Short Form Health Survey (SF-12®) [42-44] and Pain intensity (Numerical Rating Scale) and interference [45];
- Self-efficacy (or sense of confidence in activities including personal hygiene, household participation, maintaining relationships, accessing community and leisure pursuits), assessed by 16-item Moorong Self Efficacy Scale (MSES) [46];
- Indicators of social and economic participation [47], (including return to employment; relationship status, etc.) and contextual factors such as status of financial support, compensation and legal matters; and
- Survival times after injury.

Outcome measures a) to c) will be accessed soon after the start of specialist SCIU care and at 1, 3, 6 and 12 months after injury. Outcome d) will be collected from the hospital dataset. Outcomes e) to g) will be assessed by telephone follow-up at 6, 12 and 24 months post-injury event and deaths confirmed with the relevant Registry of Births, Deaths and Marriages.

### Analysis and reporting

The data will be analysed using methods conventionally employed for cohort designs, including multivariate logistic regression to test for between-group differences in outcomes for patients commencing specialist care in SCIU more than 24 hours after injury (*cf ≤* 24hours). The cross-jurisdictional nature of the research design allows analysis of effects on TSCI patient outcomes at several levels: 1) individual (e.g., SCI associated with multi-trauma or traumatic brain injury *vs* isolated SCI); 2) external cause of injury (e.g. high speed MVA *vs* low fall); 3) injury setting and remoteness (e.g. rurality); 4) triage, transport and inter-hospital transfer processes, and 5) emergency/regional/trauma hospital systems in the two States, which may interact to influence access to, and quality of, care received.

This study design will enable analyses to be conducted at the patient level and the health system level. Multivariate, multilevel regression analysis will be performed to determine patient-level factors (e.g. age, co-morbidities, socio-economic status) and hospital-level factors associated with variability (e.g. inter-hospital transfer protocols).

Results will be reported in accordance with the STROBE statement [48], which provides guidelines for reporting observational studies, to ensure that any issues concerning confounding, bias and generalizability are transparent.

### Informed consent and ethics

Patients will be informed by mail regarding their inclusion in the project, with information and an opportunity to ‘opt out’ (withdraw from the study) at that point.

Lead ethics approval for the study (including ‘opt out’ consent) was granted in NSW by Cancer Institute HREC in December 2012 and in Victoria by Austin Health HREC in September 2013. Site specific approvals have been obtained from Hunter New England Local Health District (LHD), South Western Sydney LHD, South Eastern Sydney LHD, Northern Sydney LHD, Sydney LHD, St Vincent’s Hospital, Royal Rehabilitation Hospital, NSW Ambulance, The Alfred Hospital, Royal Melbourne Hospital and Victoria Ambulance. All applications for amendments with refined versions of the Participant Information Statements, Interview and Data Dictionary to date have also been approved.

## Discussion

This project uniquely brings together multiple agencies, specialists and organisations across healthcare services in two Australian States. Further, in concert with international health system-wide analyses of patient care and flow after TSCI, this project will characterise the nature and timing of care received during the time-critical period of the early post injury patient journey. Detailed data collection will begin from the scene of injury through Ambulance Care, ED, radiology, operating theatre, ICU and/or hospital wards in district/regional hospitals, Major Trauma Service and the SCIU (acute and rehabilitation phases) to discharge and follow up.

Parallel analysis of both the detailed environmental scan of health service infrastructure and the clinical pathway data from patients, will enable investigation of associations between the quality and efficiency of healthcare delivery and health outcomes in patients with traumatic spinal cord injury. In so doing, this research will also identify factors along the treatment continuum that may influence the achievement of the best patient outcomes; including rurality, inter-hospital transfers, early commencement of specialised care, multi-trauma, altered level of consciousness, and presence of distracting injuries that can affect long-term outcomes. Emphasis will be placed on developing a comprehensive understanding of how the variation in early care pathway/s and treatment can be addressed to achieve optimal patient outcomes and reduction in economic costs to the community. This comprehensive approach aims to address national health priority issues, and equally learn from, and provide learning to other national and international performance and policy models.

Ongoing international collaborations using the methods and ACT simulation tool described earlier [33], will assist in benchmarking system performance and examining the impact of clinical pathways through different healthcare systems. The ability to simulate and measure the effect of system wide changes prior to implementation will strengthen the capacity of the findings of this research to influence policy and practice decisions for the improvement of health system performance and therefore the best management of and outcomes for those with TSCI.
